# Evaluating the educational quality and reliability of YouTube videos on red light therapy for eye diseases

**DOI:** 10.1007/s10103-026-04920-6

**Published:** 2026-06-26

**Authors:** Elvin Çelenk, Işıl Merve Torun

**Affiliations:** 1 Department of Ophthalmology, Yalova University, Yalova, Turkey; 2 Department of Ophthalmology, Sultan 2. Abdülhamid Han Eğitim ve Araştırma Hastanesi, Istanbul, Turkey

**Keywords:** Red light therapy, YouTube, Eye disease, Scoring systems

## Abstract

To investigate the use of YouTube videos as an educational and informational resource regarding the use of red light therapy in eye diseases. On May 1, 2025, a comprehensive search was conducted on the YouTube platform (https://www.youtube.com) using the keywords “Red light therapy in eye disease” and “Use of red light therapy in eye diseases for patient information”. Videos under 60 s in duration, those lacking audio, duplicate entries, irrelevant content, and videos with disabled comment sections were excluded from the analysis. These exclusion criteria were predefined prior to data collection. Following video selection (E.Ç., M.T.), all videos were independently evaluated by two ophthalmologists, and statistical analyses were performed using the finalized dataset. Parameters assessed included total views, number of comments, likes, time elapsed since upload, video duration, view rate, interaction index, video source, and content characteristics. In addition, videos were categorized according to quality level based on Global Quality Score (GQS) scores as low-to-moderate quality (GQS < 4) and high quality (GQS ≥ 4). Comparisons were then performed according to quality category with respect to uploader source, video purpose, and engagement-related parameters. Out of 110 YouTube videos reviewed, 53 were excluded based on predefined criteria. The remaining 57 videos covered myopia (22.8%), dry eye (33.3%), diabetic retinopathy (12.3%), and age-related macular degeneration (31.6%). Inter-rater agreement was high (mDISCERN ICC: 0.952; JAMA ICC: 0.923; GQS ICC: 0.890). Videos uploaded by physicians had significantly higher mDISCERN, JAMA, and GQS scores. Educational videos had higher mDISCERN and JAMA scores than patient-information videos. A moderate positive correlation was found between the number of likes and the interaction index (*r* = 0.458, *p* < 0.01). The interaction index was not significantly associated with mDISCERN, JAMA, or GQS scores, indicating that engagement metrics were not reliable indicators of informational quality. Among disease categories, age-related macular degeneration videos had the highest mean quality scores, although the differences were not statistically significant. Most videos were classified as low-to-moderate quality (82.5%), while 17.5% were classified as high quality. High-quality videos had significantly higher view counts and like counts than low-to-moderate quality videos. The overall quality and formal reliability of YouTube videos on red light therapy in ophthalmology were low to moderate. Physician-uploaded and educational videos provided higher-quality information. Although high-quality videos showed greater engagement, popularity metrics were not reliable indicators of informational quality. More evidence-based and accessible video content is needed to support patient education.

## Introduction

Recently, the use of the Internet by patients has become an important avenue for accessing health information. While the ease of rapid access offers significant convenience, it is crucial that the accuracy and reliability of the information accessed are controllable. For patients seeking information online, the validity and trustworthiness of health-related content should be critically evaluated by experts in the field. Although online resources provide valuable access to information, they inherently carry risks because of the potential presence of misleading or harmful content. Therefore, establishing appropriate standards for these accessible environments is essential. Without such standards, patients may encounter risks that hinder their ability to make informed decisions [1].

YouTube, a widely used platform characterized by ease of use and open access, where individuals can share videos on various topics, ranks as the second most visited website worldwide. YouTube videos are commonly used as information sources by patients, medical students, physicians, and other healthcare professionals. It is important to ascertain the accuracy and reliability of the information obtained from these sources [2].

Red light therapy is a treatment modality that aims to support the body’s healing processes by using low-intensity red and near-infrared light wavelengths. This therapy is also known as “low-level laser therapy” (LLLT), “photobiomodulation,” or “LED therapy.” LLLT employs specific wavelengths within the electromagnetic spectrum (630–850 nm) to stimulate cellular activity. It stimulates mitochondria, the energy source of cells, to enhance adenosine triphosphate (ATP) production and promotes the release of nitric oxide (NO) from tissues. Consequently, increased blood flow to the tissue improves nutrient and oxygen delivery, while facilitating the rapid removal of toxins. The therapy also reduces pro-inflammatory cytokines and increases anti-inflammatory cytokines while regulating cellular apoptosis. In ophthalmology, red light therapy, particularly repeated low-level red light (RLRL), has been used to treat various conditions, including age-related macular degeneration, myopia, dry eye disease, and diabetic retinopathy [3, 4].

YouTube provides unrestricted access to a wide range of health-related videos; however, these videos are not subjected to any form of peer review or quality control prior to their publication. This lack of oversight underscores the importance of systematically evaluating the reliability and accuracy of this information [5]. Misinformation, especially regarding treatment options, can lead to misunderstandings, reinforce patient biases against evidence-based therapies, and potentially undermine trust between patients and healthcare providers [6].

In ophthalmology, numerous studies have assessed the informative and educational quality of videos related to eye diseases [7, 8]. However, a systematic evaluation of the quality, reliability, and suitability of YouTube videos specifically addressing red light therapy for patient education has not been reported.

Therefore, this study aimed to assess the quality and reliability of YouTube videos related to this increasingly utilized treatment modality for ophthalmic diseases.

## Method

This cross-sectional observational study was based on a systematic analysis of publicly accessible YouTube videos. The study was conducted in accordance with the principles of the Declaration of Helsinki. As the study relied exclusively on publicly available videos and did not involve direct interaction with participants or access to identifiable private information, ethics committee approval and informed consent to participate were not required. The study design represents a structured video-based content analysis using predefined inclusion and exclusion criteria. On May 1, 2025, a comprehensive search was conducted on the YouTube platform (https://www.youtube.com) using the keywords " red light therapy in eye disease” and " use of red light therapy in eye diseases for patient information”. The platform’s default search filter, which sorts the results by relevance, was applied. To minimize personalization and search bias, the search was performed in a browser with all history and cache cleared and without logging into any user account.

The inclusion criteria were established prior to the data collection. Only English-language videos were considered for the analysis. Advertisements displayed either among the search results or at the beginning of the videos were excluded from the study. Videos with a duration of less than 60 s, those lacking audio, duplicate entries, and videos deemed irrelevant to the topic were excluded from the analysis. Videos shorter than 60 s were excluded because they were considered unlikely to provide sufficient time for structured discussion of treatment indications, benefits, risks, and limitations. Videos in which the uploader had disabled user engagement features, such as likes or comments, were also excluded. All videos were independently evaluated and scored by two experienced ophthalmologists (E.Ç. and M.T.), each working from separate locations. Because the raters could view uploader identity and video characteristics during assessment, the evaluation was considered independent and not masked. Statistical analyses were performed by E.Ç. using the finalized dataset.

Videos were classified as irrelevant if they did not discuss red light therapy within an ophthalmic context, focused exclusively on dermatological or cosmetic applications, mentioned red light therapy without substantive explanation, or consisted primarily of promotional content without educational value. This classification was performed independently by both reviewers according to predefined criteria to minimize subjective bias.

The following parameters were assessed for each video: number of views, number of comments, number of likes, time since upload (video age), video duration, like rate, view rate (views per day), interaction index, video source, and video purpose. Like rate was calculated as (number of likes / number of views) × 100. View rate was calculated as the total number of views divided by video age in days. The interaction index was calculated as [(number of likes + number of comments) / number of views] × 100. Video source was categorized consistently as physician, optometrist, patient, health-related YouTube channel, or AI-generated content. Video purpose was categorized as education or patient information. Health-related YouTube channels included non-physician medical, wellness, clinical, commercial, or educational channels that provided health-related content but were not uploaded by an identifiable physician or optometrist. AI-generated videos were identified based on explicit disclosure within the video description or narration, the presence of automated synthetic voice patterns, and the absence of identifiable human authorship. These videos were categorized separately to explore potential structural and qualitative differences. Dislike counts were not included in the final analysis because they were absent or negligible in most videos and are no longer consistently displayed publicly by YouTube. Therefore, dislike-based metrics such as the Video Power Index were not used, as they may have been disproportionately influenced by like counts rather than reflecting balanced viewer engagement. All videos were evaluated for quality and reliability using three established scoring tools: modified DISCERN, Journal of the American Medical Association (JAMA) criteria, and Global Quality Score (GQS).

A modified version of the original DISCERN questionnaire was used to evaluate the reliability of the health-related information presented in the videos. The modified DISCERN tool consists of five binary (yes/no) questions: (1) Is the video clear, concise, and easy to understand (2)? Does it utilize reliable sources of information (3) Is the content presented in a balanced and unbiased manner (4) Are additional sources or references provided for patient follow-up (5)? Are areas of uncertainty or ongoing debates acknowledged? Each “yes” response was awarded 1 point, resulting in a total possible score ranging from 0 to 5.

JAMA benchmark criteria were used to evaluate the informational quality of ‘red light therapy eye disease-related videos’ on YouTube. This assessment tool comprises four key components: authorship, attribution (citation), disclosure (description), and currency. Each criterion was scored as either 0 (not present) or 1 (present), yielding a total score ranging from 0 to 4. The original 0–4 JAMA scoring structure was retained to maintain comparability with prior YouTube-based medical video studies. Higher scores indicated greater adherence to JAMA benchmark criteria; no additional 5-point conversion was applied in the final analysis.

The overall quality of the videos was assessed using the GQS, a five-point rating scale developed by Bernard et al. to evaluate the usefulness and presentation of online health information. In this system, a score of 1 reflects very poor quality; the video lacks structure, omits most key information, and is not helpful to patients. A score of 2 indicates poor quality, although some information may be present; however, its practical value for patients remains limited. A score of 3 suggests moderate quality, with some relevant information presented in a somewhat organized manner. A score of 4 indicates good quality, meaning the content is well-structured, informative, and generally useful for patients. Finally, a score of 5 represented excellent quality, with clear and comprehensive information delivered in a well-organized and patient-friendly manner.

The Number Cruncher Statistical System (NCSS) 2007 (Kaysville, Utah, USA) program was used for statistical analyses. Descriptive statistical methods (mean, standard deviation, median, frequency, ratio, minimum, maximum) were used to evaluate the study data, and the distribution of the data was evaluated using the Shapiro-Wilk test. The Kruskal-Wallis test was used to compare three or more groups of quantitative data, and the Mann-Whitney U test was used to compare two groups. Spearman’s correlation analysis was used to determine relationships between quantitative variables, including mDISCERN, JAMA, GQS, number of views, number of likes, number of comments, video length, viewing time, viewing rate, and interaction index. Inter-rater agreement was assessed using intraclass correlation coefficients (ICCs), calculated with a two-way random-effects model for absolute agreement based on average measures. Videos were categorized according to quality level based on GQS scores as low-to-moderate quality (GQS < 4) and high quality (GQS ≥ 4), and comparisons were performed accordingly. Categorical variables were compared using Fisher’s exact test when expected cell counts were small or zero; Pearson chi-square testing was used only when its assumptions were satisfied. Statistical significance was defined as *p* < 0.05, and exact p-values were reported where possible.

## Results

In this study, 110 YouTube videos related to the topic were systematically reviewed and evaluated by two independent ophthalmologists. A high level of inter-rater agreement was observed between the two ophthalmologists (mDISCERN ICC: 0.952; JAMA ICC: 0.923; and GQS ICC: 0.890). A total of 53 videos that did not meet the inclusion criteria were excluded from the analyses.

Among the remaining videos, 13 were related to myopia (22.8%), 19 to dry eye disease (33.3%), 7 to diabetic retinopathy (12.3%), and 18 to age-related macular degeneration (31.6%).

When categorized by uploader type, among the myopia-related videos, six were uploaded by physicians, six by optometrists, and one by an AI-generated source. Of the dry eye videos, 10 were uploaded by physicians, 3 by optometrists, 2 by patients, 3 by health-related channels, and 1 by an AI-generated source. In the diabetic retinopathy group, two videos were uploaded by physicians, one by an optometrist, one by a patient, one by a health-related YouTube channel, and two by AI-generated sources. Regarding age-related macular degeneration, 16 videos were uploaded by physicians and two by optometrists.

Regarding the purpose of upload, nine of the myopia-related videos aimed to inform patients, while four were educational. Among the dry eye videos, 15 were categorized as patient-information videos and four as educational videos. Six diabetic retinopathy videos were categorized as patient-information videos, and one was categorized as an educational video. In the case of age-related macular degeneration, 14 were categorized as patient-information videos and four as educational videos.

The mean values of the video metrics are presented in Table 1, and the average scores for mDISCERN, JAMA, and GQS according to observers are shown in Table 2. When videos were categorized according to practical quality thresholds, only a limited proportion met high-quality criteria (GQS ≥ 4). The majority of videos were classified as low to moderate quality, reinforcing the overall findings of insufficient educational reliability. Videos uploaded by physicians had significantly higher mDISCERN (*p* = 0.02), JAMA (*p* = 0.001), and GQS (*p* = 0.001) scores than those uploaded by other sources. Additionally, the mDISCERN score of optometrist-uploaded videos was significantly higher than that of videos from health-related channels (*p* = 0.001). However, in terms of the interaction index, optometrist videos showed significantly lower values than those from health channels (*p* = 0.001).

**Table 1:** Descriptive statistics of video metrics

Variable	Mean ± SD	Median (Q1–Q3)
Number of views	3,854,829.05 ± 12,279,560.89	1,498 (262–1,000,000)
Number of likes	161,716.37 ± 491,287.89	31 (1–267)
Number of comments	319.54 ± 985.89	2 (0–39)
Video length (min)	10.47 ± 11.95	7.36 (3.55–12.41)
Video age (days)	577.09 ± 673.86	305 (120–730)
View rate (views/day)	10,256.60 ± 36,346.91	5.52 (0.82–2,352.94)
Interaction index	4.61 ± 9.67	2.37 (0.66–3.25)

Values are presented as mean ± SD and median (Q1–Q3). Dislike counts and Video Power Index (VPI) analyses were excluded from the final analysis because dislike counts were absent/negligible in most videos and were not consistently publicly available

Abbreviations: *min,* minutes; *SD,* standard deviation; *Q1,* first quartile; *Q3,* third quartile

**Table 2:** Comparison of scoring measurements according to observers

Score	Observer 1 Mean ± SD; Min–Max (Median)	Observer 2 Mean ± SD; Min–Max (Median)	*p*	ICC
mDISCERN	2.76 ± 1.22; 1–5 (2)	2.85 ± 1.04; 1–5 (3)	0.276	0.952
JAMA	2.98 ± 1.01; 1–4 (3)	2.95 ± 1.03; 1–4 (3)	0.033*	0.923
GQS	2.85 ± 1.04; 1–5 (3)	2.78 ± 1.10; 1–5 (3)	0.001*	0.890

Wilcoxon test was used. *p < 0.05

Abbreviations: *mDISCERN,* modified DISCERN; *JAMA,* Journal of the American Medical Association; *GQS,* Global Quality Score; *Min,* minimum; *Max,* maximum; *SD,* standard deviation; *ICC,* intraclass correlation coefficient

Videos uploaded for educational purposes scored significantly higher in mDISCERN and JAMA than those intended for patient information (*p* = 0.021 and *p* = 0.016, respectively). A statistically significant moderate positive correlation was observed between the number of likes and the interaction index (*r* = 0.458, *p* < 0.01). No dislike-based Video Power Index analysis was reported because dislike counts were absent or negligible in most videos and were not consistently available through the public YouTube interface.

The correlation analysis between video metrics and evaluation scores is presented in Table 3, and the distribution of scores according to disease groups is shown in Table 4.

**Table 3:** Correlation analysis between video metrics and evaluation scores

Variable		1	2	3	4	5	6	7	8	9	10
1. Number of views	r	1									
	p										
2. Number of likes	r	.897**	1								
	p	.000	.								
3. Number of comments	r	.818**	.826**	1							
	p	.000	.000	.							
4. Video length	r	.575**	.481**	.507**	1						
	p	.000	.000	.000	.						
5. Video age	r	.463**	.369**	.289*	.200	1					
	p	.000	.005	.029	.139	.					
6. View rate	r	.912**	.832**	.798**	.535**	.113	1				
	p	.000	.000	.000	.000	.404	.				
7. Interaction index	r	.136	.458**	.394**	.045	-.243	.244	1			
	p	.313	.000	.002	.743	.068	.068	.			
8. mDISCERN_Avg	r	.441**	.292*	.400**	.454**	.204	.417**	-.025	1		
	p	.001	.029	.002	.000	.131	.001	.854	.		
9. JAMA_Avg	r	.514**	.417**	.442**	.496**	.306*	.436**	.046	.700**	1	
	p	.000	.001	.001	.000	.022	.001	.734	.000	.	
10. GQS_Avg	r	.519**	.401**	.481**	.371**	.285*	.478**	-.006	.759**	.765**	1
	p	.000	.002	.000	.005	.033	.000	.963	.000	.000	.

Spearman correlation analysis. *p < 0.05, **p < 0.01. Video Power Index (VPI) was removed from the final analysis

Abbreviations: *mDISCERN,* modified DISCERN; *JAMA,* Journal of the American Medical Association; *GQS,* Global Quality Score; *Avg,* average

**Table 4:** Distribution of scores according to disease groups

Score	Disease group	*n*	Mean ± SD	Median (Q1–Q3)	*p*
mDISCERN	AMD	18	3.06 ± 1.20	3 (2–4)	0.957
	DRP	7	2.79 ± 1.60	2 (2–4)	
	Dry eye	19	2.89 ± 1.59	3 (1–4.25)	
	Myopia	13	2.92 ± 1.38	2 (2–4)	
JAMA	AMD	18	3.26 ± 0.81	3.5 (3–4)	0.089
	DRP	7	2.64 ± 1.18	3 (2–3.25)	
	Dry eye	19	2.82 ± 0.93	3 (2.25–3.5)	
	Myopia	13	2.65 ± 0.63	3 (2.5–3)	
GQS	AMD	18	2.91 ± 0.69	2.5 (2.5–3.5)	0.360
	DRP	7	2.57 ± 0.93	2.5 (2.25–3)	
	Dry eye	19	2.45 ± 0.83	2.5 (1.75–3)	
	Myopia	13	2.46 ± 0.83	2.5 (2–3)	

Kruskal-Wallis test was used. Values are presented as mean ± SD and median (Q1–Q3)

Abbreviations: *AMD,* age-related macular degeneration; *DRP,* diabetic retinopathy; *mDISCERN,* modified DISCERN; *JAMA,* Journal of the American Medical Association; *GQS,* Global Quality Score; *n,* number; *SD,* standard deviation; *Q1,* first quartile; *Q3,* third quartile

Although age-related macular degeneration videos showed relatively higher mean quality scores, subgroup differences did not reach statistical significance (Table 4), suggesting variability in content quality across disease categories.

When videos were categorized according to practical quality thresholds, 47 videos (82.5%) were classified as low-to-moderate quality (GQS < 4) and 10 videos (17.5%) as high quality (GQS ≥ 4). According to uploader type, 10 of 37 physician-uploaded videos (27.0%) were classified as high quality, whereas no high-quality videos were identified among patient-uploaded, optometrist-uploaded, health-channel, or AI-generated videos. Because several uploader categories contained small sample sizes and zero-frequency cells, Fisher’s exact test was used for this comparison; the relationship between uploader type and quality category was not statistically significant (*p* = 0.149) (Table 5).

**Table 5:** Quality evaluation according to uploader type

Uploader type	Low-to-moderate quality (GQS < 4)	High quality (GQS ≥ 4)	*p*
Physician	27 (73.0%)	10 (27.0%)	0.149
Patient	2 (100%)	0 (0%)	
Optometrist	8 (100%)	0 (0%)	
Health-related channel	6 (100%)	0 (0%)	
AI-generated content	4 (100%)	0 (0%)	

Fisher’s exact test was used because several cells had small expected counts and zero frequencies. *p* < 0.05 was considered statistically significant. Percentages are calculated within each uploader type

Abbreviations: *GQS,* Global Quality Score; *AI,* artificial intelligence

When quality category was compared according to video purpose, 2 of 13 educational videos (15.4%) and 8 of 44 patient-information videos (18.2%) were classified as high quality. No statistically significant association was found between video purpose and quality category (*p* = 0.791) (Table 6).

**Table 6:** Quality evaluation according to video purpose

Aim of the video	Low-to-moderate quality (GQS < 4)	High quality (GQS ≥ 4)	Total	*p*
Education	11 (84.6%)	2 (15.4%)	13 (100%)	0.791
Patient information	36 (81.8%)	8 (18.2%)	44 (100%)	
Total	47 (82.5%)	10 (17.5%)	57 (100%)	

Fisher’s exact test was used. *p* < 0.05 was considered statistically significant. Percentages are calculated within each row

Abbreviations: *GQS*: Global Quality Score

When engagement-related parameters were compared according to quality category, videos in the high-quality group (GQS ≥ 4) had significantly higher view counts and like counts than those in the low-to-moderate quality group (GQS < 4) (Table 7).

**Table 7:** View count and like count according to video quality category

Metric	Video quality category	*n*	Mean ± SD	Median (Q1–Q3)	*p*
View count	Low-to-moderate quality (GQS < 4)	47	342,508.87 ± 822,409.34	972 (140.5–12,496.5)	0.001**
	High quality (GQS ≥ 4)	10	20,362,733.90 ± 23,823,597.90	12,250,000 (288,624–36,000,000)	
Like count	Low-to-moderate quality (GQS < 4)	47	68,600.36 ± 305,470.45	20 (1–105)	0.004**
	High quality (GQS ≥ 4)	10	599,361.60 ± 875,668.62	23,829 (73.25–1,114,021.5)	

Mann-Whitney U test was used. **p < 0.01. Values are presented as mean ± SD and median (Q1–Q3). Video Power Index (VPI) was removed from this table because dislike counts were absent/negligible in most videos and were not consistently publicly displayed by YouTube

Abbreviations: *GQS,* Global Quality Score; *SD,* standard deviation; *Q1,* first quartile; *Q3,* third quartile; *n,* number

## Discussion

Red light therapy, particularly in the form of low-level red light (RLRL), has recently attracted attention as a non-invasive treatment for various ophthalmic conditions, including myopia, dry eye disease, diabetic retinopathy, and age-related macular degeneration (AMD) [4, 7, 8] It is important to acknowledge that the ophthalmic conditions included in this analysis differ substantially in pathophysiology, evidence base, and regulatory status with respect to red light therapy. These conditions were analyzed collectively because they represent the most frequently discussed indications on YouTube; however, differences in scientific support, clinical acceptance, and regulatory status may partially explain variations in video quality. The regulatory status of red light therapy differs across ophthalmic applications. Some uses may have regulatory authorization under specific clinical conditions, whereas others remain investigational or are applied off-label. This distinction is relevant when interpreting YouTube content, because approved indications are more likely to be discussed in evidence-based educational contexts, while investigational or off-label applications may generate more heterogeneous, commercially driven, or speculative material. Therefore, regulatory status may influence both the availability and the quality of online video content.

RLRL therapy generally involves exposing the eyes to low-intensity red light using desktop-based devices for short periods multiple times per day. Current evidence suggests that this approach may effectively slow axial elongation and associated refractive changes in children and adolescents with progressive myopia. In addition, YouTube-based studies on ophthalmic diseases have previously evaluated the reliability and educational quality of videos related to age-related macular degeneration and dry eye disease. These findings indicate that ophthalmology-related video content may vary considerably in quality and reliability, supporting the need for topic-spesific evaluations such as the present study.

 [7, 8] In light of these recent developments and the growing interest in RLRL, patients frequently seek additional information through digital platforms such as YouTube. However, to the best of our knowledge, there is a lack of studies specifically evaluating the quality and reliability of YouTube videos addressing this therapeutic modality. Therefore, the present study aimed to evaluate the content, source, and quality of YouTube videos related to RLRL using standardized assessment tools.

The present findings are consistent with those of Kesimal et al., who evaluated YouTube videos as information sources for optic neuritis and reported that the overall video quality was suboptimal. Their findings indicated that videos produced by physicians received higher scores on the JAMA and GQS scales, underscoring the pivotal role of expert authorship in generating reliable online health content. Furthermore, they observed no substantial correlation between video metrics such as views, likes, or comments and content quality, highlighting the need for validated and objective assessment tools in future studies [9]. Similarly, in the present study, the interaction index was not significantly associated with mDISCERN, JAMA, or GQS scores. These findings emphasize the importance of using standardized and reliable tools when evaluating the quality of online video content rather than relying solely on popularity-related metrics.

Engagement-related metrics, including views, likes, comments, view rate, and interaction index, may be influenced by multiple external factors such as video age, algorithmic amplification, and platform-specific recommendation systems. Although view rate was calculated to partially adjust for temporal exposure, these metrics should be interpreted cautiously and should not be considered direct proxies for informational quality. In addition, dislike counts could not be reliably incorporated because they were absent or negligible in most videos and are no longer consistently displayed through the public YouTube interface. Therefore, correlations involving popularity-related variables do not imply causal relationships.

In a related study, Khalil et al. reported that patient-generated videos on aortic stenosis had viewership and engagement metrics comparable to those of professional content, despite the latter being more reliable [10]. In line with these findings, our study observed that videos narrated by patients received higher view counts than those uploaded by other sources. Although medical experts possess greater subject knowledge, they may have difficulty conveying complex information in a manner that is easily understandable to non-professionals. The use of specialized terminology and complex language structures in professionally produced videos may reduce accessibility for the general public, which could partly explain the higher engagement observed with patient-narrated content. Nevertheless, in terms of likes and comments, videos created by physicians still demonstrated higher average metrics than other sources. Moreover, videos uploaded by physicians and optometrists had significantly higher mean scores on the mDISCERN, JAMA, and GQS scales, reaffirming their superior informational value.

AI-generated videos showed a more standardized narration style, limited identifiable authorship, and less discussion of clinical uncertainty than physician-produced videos. Although some were clearly organized, the absence of transparent authorship and source citation may reduce accountability and limit their educational value.

The findings of this study are also consistent with those of Kocamış et al., who evaluated YouTube videos on corneal cross-linking and concluded that although the platform may serve as an information source, content quality varies considerably, necessitating user discretion. They also emphasized that videos originating from recognized institutions or medical professionals are generally more reliable and cautioned against using popularity metrics as sole indicators of credibility [11]. In a similar vein, Garip et al. found that videos uploaded by physicians on ptosis surgery were of significantly higher quality than those uploaded by private clinics [12]. Our results support these observations, as physician-uploaded content showed higher JAMA and GQS scores, further highlighting the value of professional authorship in online medical education.

Sayin O. et al. assessed YouTube videos related to vitreoretinal surgery and concluded that most were of poor quality and reliability, recommending caution when such content is used for educational purposes [13]. These findings are consistent with the present study, which similarly found the overall quality of RLRL-related videos to be low to moderate. Viewers who use YouTube as an educational resource should therefore critically evaluate the source, authorship, and supporting evidence of each video.

Karacan G. et al. analyzed videos uploaded by healthcare professionals regarding botulinum toxin therapy for migraine and reported high-quality scores despite relatively low viewer engagement. They also noted that some videos, despite appearing informative, had the potential to mislead viewers [14]. In the present study, videos related to AMD showed higher mean mDISCERN, JAMA, and GQS scores compared with other subgroups, although this difference did not reach statistical significance. This pattern may partly reflect differences in the level of regulatory acceptance and clinical evidence across indications, which could contribute to higher production quality and more evidence-based presentations for certain topics.

When interpreted by disease category, some subgroup-level differences were observed. Videos related to age-related macular degeneration tended to show relatively higher quality scores, which may reflect comparatively greater clinical maturity and regulatory recognition for this indication [15, 16]. In contrast, videos on myopia and dry eye disease appeared more heterogeneous in both source and informational quality, likely reflecting differences in clinical adoption and the evolving evidence base [17, 18]. For diabetic retinopathy-related content, the more limited and mixed evidence base may also have contributed to less consistent presentation patterns [19]. Although these subgroup differences did not reach statistical significance, they suggest that disease-specific context may influence the quality and characteristics of YouTube content.

As demonstrated by Mangan S. et al., videos uploaded by academic institutions for educational purposes tend to have higher quality and reliability scores than those uploaded for other purposes [20]. Consistent with this observation, educational videos in the present study scored significantly higher than patient-information videos on the mDISCERN and JAMA assessments (*p* = 0.021 and *p* = 0.016, respectively).

From an ethical and educational perspective, AI-generated medical content raises important concerns regarding transparency, accountability, and the potential dissemination of oversimplified or non-peer-reviewed information. The absence of clearly identifiable authorship may limit responsibility for content accuracy. As AI-based content production becomes increasingly prevalent, clear disclosure of AI involvement and adherence to evidence-based standards will be essential for maintaining public trust and safeguarding patient education. However, the present study did not aim to technically verify whether AI tools were used; classification was based only on observable video characteristics and explicit disclosure.

This study had several limitations. First, only English-language videos were included, which may limit the generalizability of our results. Future studies should include videos in other languages to provide a more comprehensive understanding of the subject. Second, YouTube is a dynamic platform where content is continuously added, removed, or reordered, which may influence results over time. Third, search results may vary depending on keyword phrasing and geographic location. Although predefined criteria were used to classify irrelevant videos, some degree of subjective judgment may still have influenced video selection, which could affect reproducibility and introduce selection bias. In addition, although view rate was calculated to partially account for temporal exposure, engagement metrics remain influenced by platform algorithms and recommendation systems, limiting the interpretability of correlations involving popularity-related findings. Dislike counts could not be reliably incorporated because they were absent or negligible in most videos and are no longer consistently available through the public YouTube interface; therefore, dislike-based metrics such as the Video Power Index were excluded from the final analysis. Furthermore, mDISCERN, JAMA, and GQS evaluate formal reliability, transparency, structure, and educational quality, but they do not provide a direct systematic fact-check of each medical claim. For this reason, the present findings should be interpreted as an assessment of formal quality and reliability rather than a definitive evaluation of factual accuracy or misinformation. Future studies should incorporate evidence-based claim-level coding to distinguish inaccurate, unsupported, misleading, exaggerated, off-label, and speculative statements. Furthermore, video assessment could not be masked because uploader identity and visible video characteristics were apparent during evaluation; however, videos were independently scored by two experienced ophthalmologists, and inter-rater reliability was high. Despite the use of validated scoring systems, quality assessment inherently involves some degree of subjective interpretation. The cross-sectional design also reflects only a single time-point snapshot, whereas YouTube content may evolve over time. Nevertheless, evaluation by two independent ophthalmologists using three validated scoring systems represents a major strength of the study, with strong inter-rater reliability observed.

Professional ophthalmological societies and academic institutions should consider developing standardized, evidence-based multimedia content tailored to patient comprehension levels. Institutional verification markers and clearer disclosure standards may further enhance the reliability of online medical information platforms.

In conclusion, the reliability and formal educational quality of YouTube videos related to red light therapy vary according to disease context, although the overall quality tends to be low to moderate. Differences in evidence base, regulatory status, and clinical acceptance across indications may influence both the quantity and quality of available video content. Because popularity metrics do not reliably indicate informational quality or factual accuracy, patients should be guided toward evidence-based content produced or reviewed by qualified professionals. There is a clear need for the production of more comprehensive, high-quality, and reliable videos to support patient education regarding this emerging therapeutic approach.

## Data Availability

Publicly available data has been used.

## Abbreviations

LLLT: Low-Level Laser Therapy
ATP: Adenosine Triphosphate
NO: Nitric Oxide
RLRL: Repeated Low-Level Red Light
mDISCERN: Modified DISCERN
JAMA: Journal of the American Medical Association
GQS: Global Quality Score
